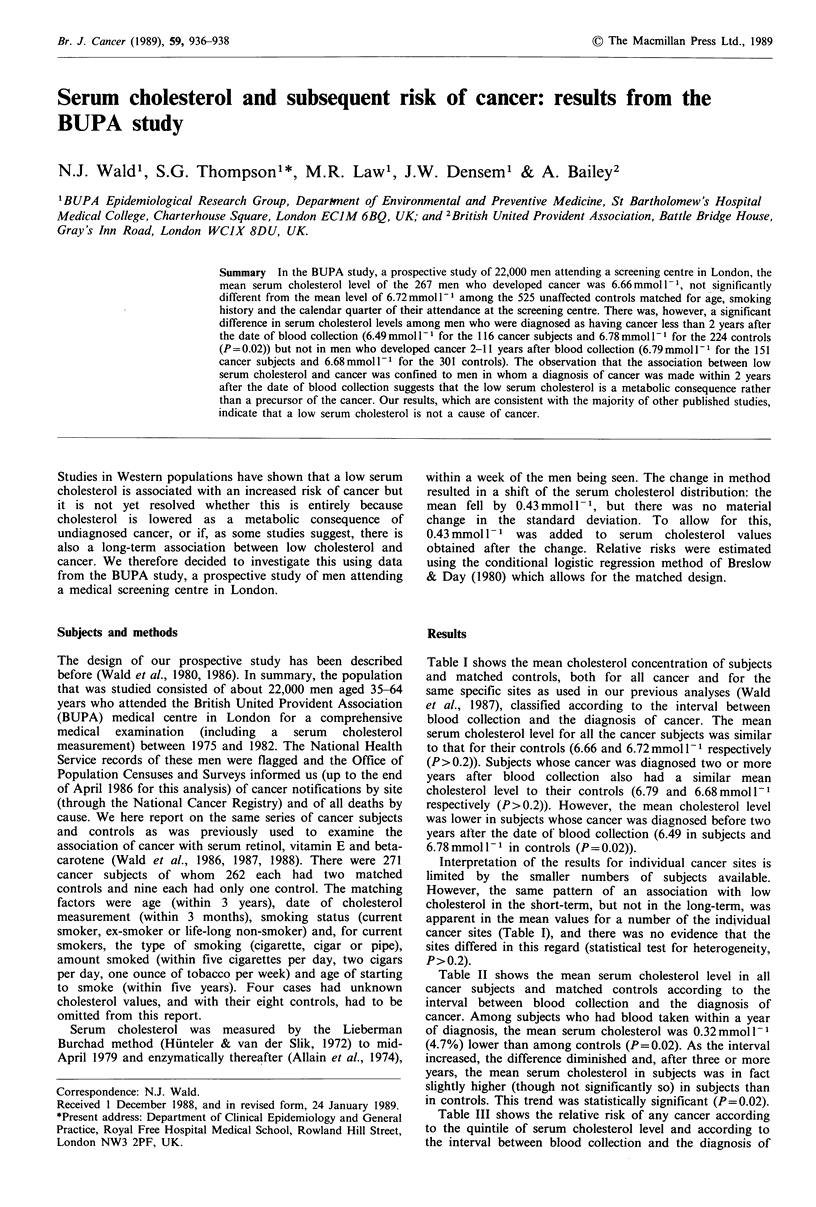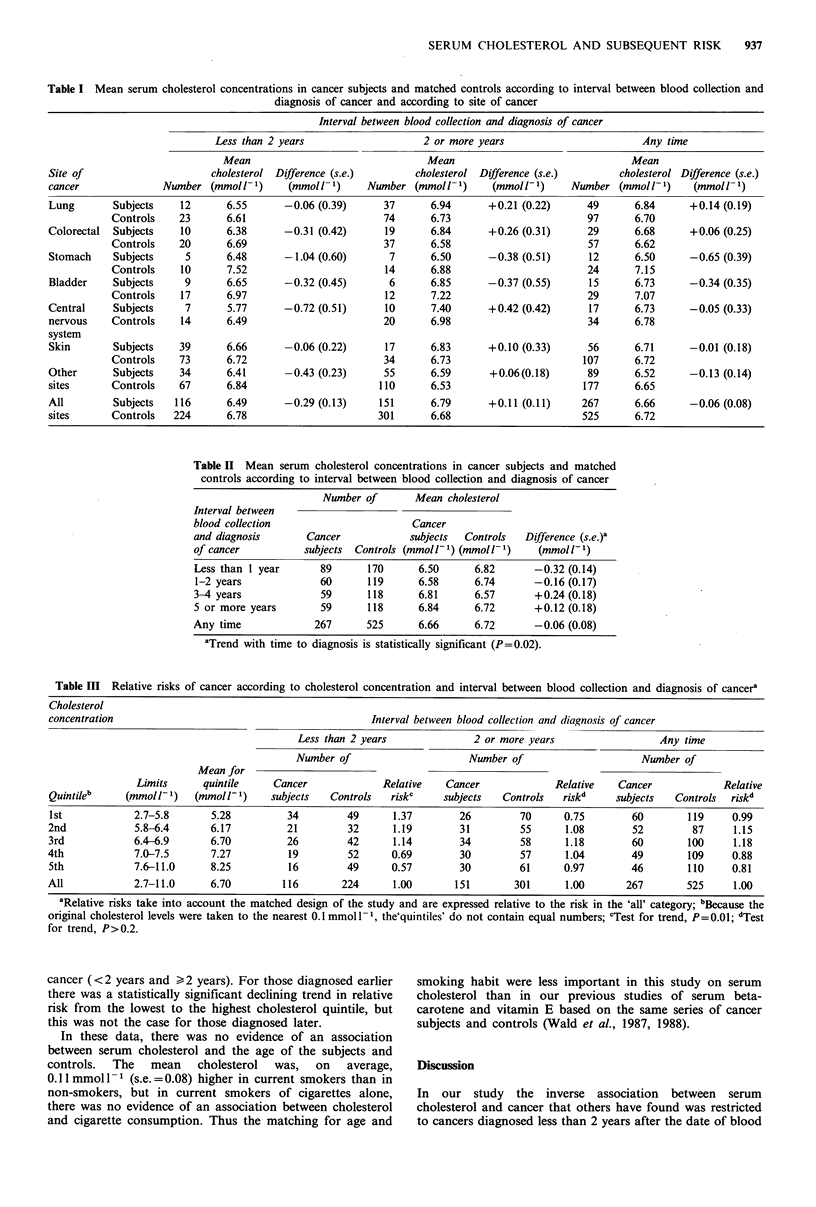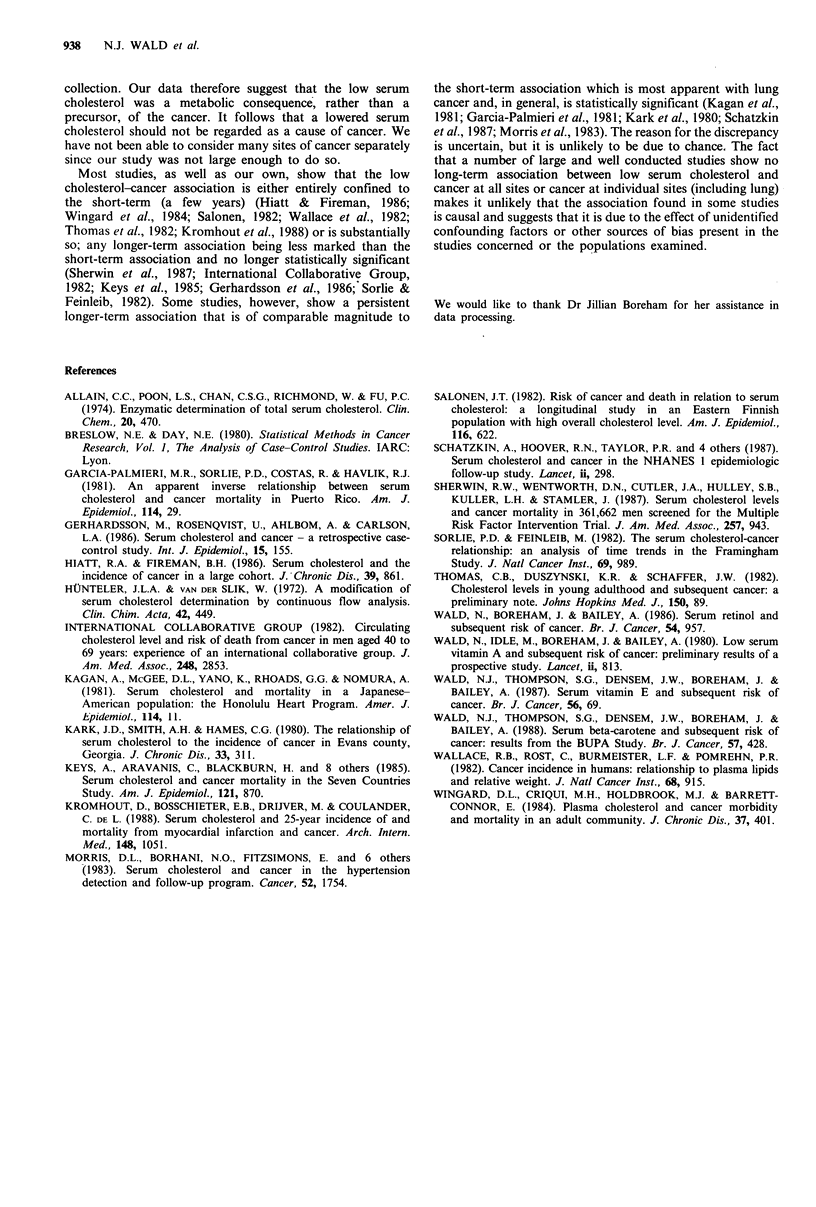# Serum cholesterol and subsequent risk of cancer: results from the BUPA study.

**DOI:** 10.1038/bjc.1989.198

**Published:** 1989-06

**Authors:** N. J. Wald, S. G. Thompson, M. R. Law, J. W. Densem, A. Bailey

**Affiliations:** Department of Environmental and Preventive Medicine, St Bartholomew's Hospital Medical College, London, UK.

## Abstract

In the BUPA study, a prospective study of 22,000 men attending a screening centre in London, the mean serum cholesterol level of the 267 men who developed cancer was 6.66 mmol l-1, not significantly different from the mean level of 6.72 mmol l-1 among the 525 unaffected controls matched for age, smoking history and the calendar quarter of their attendance at the screening centre. There was, however, a significant difference in serum cholesterol levels among men who were diagnosed as having cancer less than 2 years after the date of blood collection (6.49 mmol l-1 for the 116 cancer subjects and 6.78 mmol l-1 for the 224 controls (P = 0.02)) but not in men who developed cancer 2-11 years after blood collection (6.79 mmol l-1 for the 151 cancer subjects and 6.68 mmol l-1 for the 301 controls). The observation that the association between low serum cholesterol and cancer was confined to men in whom a diagnosis of cancer was made within 2 years after the date of blood collection suggests that the low serum cholesterol is a metabolic consequence rather than a precursor of the cancer. Our results, which are consistent with the majority of other published studies, indicate that a low serum cholesterol is not a cause of cancer.


*Br. J. Cancer* (1989), **59**, 936–938 © The Macmillan Press Ltd., 1989

### Serum cholesterol and subsequent risk of cancer: results from the BUPA study

N.J. Wald[1], S.G. Thompson[1*], M.R. Law[1], J.W. Densem[1] & A. Bailey[2]

[1]*BUPA Epidemiological Research Group, Department of Environmental and Preventive Medicine, St Bartholomew's Hospital Medical College, Charterhouse Square, London EC1M 6BQ, UK; and* [2]*British United Provident Association, Battle Bridge House, Gray's Inn Road, London WC1X 8DU, UK.*

**Summary** In the BUPA study, a prospective study of 22,000 men attending a screening centre in London, the mean serum cholesterol level of the 267 men who developed cancer was 6.66 mmol $l^{-1}$, not significantly different from the mean level of 6.72 mmol $l^{-1}$ among the 525 unaffected controls matched for age, smoking history and the calendar quarter of their attendance at the screening centre. There was, however, a significant difference in serum cholesterol levels among men who were diagnosed as having cancer less than 2 years after the date of blood collection (6.49 mmol $l^{-1}$ for the 116 cancer subjects and 6.78 mmol $l^{-1}$ for the 224 controls ($P=0.02$)) but not in men who developed cancer 2–11 years after blood collection (6.79 mmol $l^{-1}$ for the 151 cancer subjects and 6.68 mmol $l^{-1}$ for the 301 controls). The observation that the association between low serum cholesterol and cancer was confined to men in whom a diagnosis of cancer was made within 2 years after the date of blood collection suggests that the low serum cholesterol is a metabolic consequence rather than a precursor of the cancer. Our results, which are consistent with the majority of other published studies, indicate that a low serum cholesterol is not a cause of cancer.

Studies in Western populations have shown that a low serum cholesterol is associated with an increased risk of cancer but it is not yet resolved whether this is entirely because cholesterol is lowered as a metabolic consequence of undiagnosed cancer, or if, as some studies suggest, there is also a long-term association between low cholesterol and cancer. We therefore decided to investigate this using data from the BUPA study, a prospective study of men attending a medical screening centre in London.

### Subjects and methods

The design of our prospective study has been described before (Wald *et al.*, 1980, 1986). In summary, the population that was studied consisted of about 22,000 men aged 35–64 years who attended the British United Provident Association (BUPA) medical centre in London for a comprehensive medical examination (including a serum cholesterol measurement) between 1975 and 1982. The National Health Service records of these men were flagged and the Office of Population Censuses and Surveys informed us (up to the end of April 1986 for this analysis) of cancer notifications by site (through the National Cancer Registry) and of all deaths by cause. We here report on the same series of cancer subjects and controls as was previously used to examine the association of cancer with serum retinol, vitamin E and beta-carotene (Wald *et al.*, 1986, 1987, 1988). There were 271 cancer subjects of whom 262 each had two matched controls and nine each had only one control. The matching factors were age (within 3 years), date of cholesterol measurement (within 3 months), smoking status (current smoker, ex-smoker or life-long non-smoker) and, for current smokers, the type of smoking (cigarette, cigar or pipe), amount smoked (within five cigarettes per day, two cigars per day, one ounce of tobacco per week) and age of starting to smoke (within five years). Four cases had unknown cholesterol values, and with their eight controls, had to be omitted from this report.

Serum cholesterol was measured by the Lieberman Burchad method (Hünteler & van der Slik, 1972) to mid-April 1979 and enzymatically thereafter (Allain *et al.*, 1974), within a week of the men being seen. The change in method resulted in a shift of the serum cholesterol distribution: the mean fell by 0.43 mmol $l^{-1}$, but there was no material change in the standard deviation. To allow for this, 0.43 mmol $l^{-1}$ was added to serum cholesterol values obtained after the change. Relative risks were estimated using the conditional logistic regression method of Breslow & Day (1980) which allows for the matched design.

### Results

Table I shows the mean cholesterol concentration of subjects and matched controls, both for all cancer and for the same specific sites as used in our previous analyses (Wald *et al.*, 1987), classified according to the interval between blood collection and the diagnosis of cancer. The mean serum cholesterol level for all the cancer subjects was similar to that for their controls (6.66 and 6.72 mmol $l^{-1}$ respectively ($P>0.2$)). Subjects whose cancer was diagnosed two or more years after blood collection also had a similar mean cholesterol level to their controls (6.79 and 6.68 mmol $l^{-1}$ respectively ($P>0.2$)). However, the mean cholesterol level was lower in subjects whose cancer was diagnosed before two years after the date of blood collection (6.49 in subjects and 6.78 mmol $l^{-1}$ in controls ($P=0.02$)).

Interpretation of the results for individual cancer sites is limited by the smaller numbers of subjects available. However, the same pattern of an association with low cholesterol in the short-term, but not in the long-term, was apparent in the mean values for a number of the individual cancer sites (Table I), and there was no evidence that the sites differed in this regard (statistical test for heterogeneity, $P>0.2$).

Table II shows the mean serum cholesterol level in all cancer subjects and matched controls according to the interval between blood collection and the diagnosis of cancer. Among subjects who had blood taken within a year of diagnosis, the mean serum cholesterol was 0.32 mmol $l^{-1}$ (4.7%) lower than among controls ($P=0.02$). As the interval increased, the difference diminished and, after three or more years, the mean serum cholesterol in subjects was in fact slightly higher (though not significantly so) in subjects than in controls. This trend was statistically significant ($P=0.02$).

Table III shows the relative risk of any cancer according to the quintile of serum cholesterol level and according to the interval between blood collection and the diagnosis of

Correspondence: N.J. Wald.
Received 1 December 1988, and in revised form, 24 January 1989.
*Present address: Department of Clinical Epidemiology and General Practice, Royal Free Hospital Medical School, Rowland Hill Street, London NW3 2PF, UK.

**Table I** Mean serum cholesterol concentrations in cancer subjects and matched controls according to interval between blood collection and diagnosis of cancer and according to site of cancer

| | | *Interval between blood collection and diagnosis of cancer* | | | | | | | | |
|---|---|---|---|---|---|---|---|---|---|---|
| | | *Less than 2 years* | | | *2 or more years* | | | *Any time* | | |
| *Site of cancer* | | *Number* | *Mean cholesterol (mmol l$^{-1}$)* | *Difference (s.e.) (mmol l$^{-1}$)* | *Number* | *Mean cholesterol (mmol l$^{-1}$)* | *Difference (s.e.) (mmol l$^{-1}$)* | *Number* | *Mean cholesterol (mmol l$^{-1}$)* | *Difference (s.e.) (mmol l$^{-1}$)* |
| Lung | Subjects | 12 | 6.55 | −0.06 (0.39) | 37 | 6.94 | +0.21 (0.22) | 49 | 6.84 | +0.14 (0.19) |
| | Controls | 23 | 6.61 | | 74 | 6.73 | | 97 | 6.70 | |
| Colorectal | Subjects | 10 | 6.38 | −0.31 (0.42) | 19 | 6.84 | +0.26 (0.31) | 29 | 6.68 | +0.06 (0.25) |
| | Controls | 20 | 6.69 | | 37 | 6.58 | | 57 | 6.62 | |
| Stomach | Subjects | 5 | 6.48 | −1.04 (0.60) | 7 | 6.50 | −0.38 (0.51) | 12 | 6.50 | −0.65 (0.39) |
| | Controls | 10 | 7.52 | | 14 | 6.88 | | 24 | 7.15 | |
| Bladder | Subjects | 9 | 6.65 | −0.32 (0.45) | 6 | 6.85 | −0.37 (0.55) | 15 | 6.73 | −0.34 (0.35) |
| | Controls | 17 | 6.97 | | 12 | 7.22 | | 29 | 7.07 | |
| Central nervous system | Subjects | 7 | 5.77 | −0.72 (0.51) | 10 | 7.40 | +0.42 (0.42) | 17 | 6.73 | −0.05 (0.33) |
| | Controls | 14 | 6.49 | | 20 | 6.98 | | 34 | 6.78 | |
| Skin | Subjects | 39 | 6.66 | −0.06 (0.22) | 17 | 6.83 | +0.10 (0.33) | 56 | 6.71 | −0.01 (0.18) |
| | Controls | 73 | 6.72 | | 34 | 6.73 | | 107 | 6.72 | |
| Other sites | Subjects | 34 | 6.41 | −0.43 (0.23) | 55 | 6.59 | +0.06 (0.18) | 89 | 6.52 | −0.13 (0.14) |
| | Controls | 67 | 6.84 | | 110 | 6.53 | | 177 | 6.65 | |
| All sites | Subjects | 116 | 6.49 | −0.29 (0.13) | 151 | 6.79 | +0.11 (0.11) | 267 | 6.66 | −0.06 (0.08) |
| | Controls | 224 | 6.78 | | 301 | 6.68 | | 525 | 6.72 | |

**Table II** Mean serum cholesterol concentrations in cancer subjects and matched controls according to interval between blood collection and diagnosis of cancer

| *Interval between blood collection and diagnosis of cancer* | *Number of* | | *Mean cholesterol* | | |
|---|---|---|---|---|---|
| | *Cancer subjects* | *Controls* | *Cancer subjects (mmol l$^{-1}$)* | *Controls (mmol l$^{-1}$)* | *Difference (s.e.)*[a] *(mmol l$^{-1}$)* |
| Less than 1 year | 89 | 170 | 6.50 | 6.82 | −0.32 (0.14) |
| 1–2 years | 60 | 119 | 6.58 | 6.74 | −0.16 (0.17) |
| 3–4 years | 59 | 118 | 6.81 | 6.57 | +0.24 (0.18) |
| 5 or more years | 59 | 118 | 6.84 | 6.72 | +0.12 (0.18) |
| Any time | 267 | 525 | 6.66 | 6.72 | −0.06 (0.08) |

[a]Trend with time to diagnosis is statistically significant ($P=0.02$).

**Table III** Relative risks of cancer according to cholesterol concentration and interval between blood collection and diagnosis of cancer[a]

| *Cholesterol concentration* | | | *Interval between blood collection and diagnosis of cancer* | | | | | | | | |
|---|---|---|---|---|---|---|---|---|---|---|---|
| | | | *Less than 2 years* | | | *2 or more years* | | | *Any time* | | |
| | | | *Number of* | | | *Number of* | | | *Number of* | | |
| *Quintile*[b] | *Limits (mmol l$^{-1}$)* | *Mean for quintile (mmol l$^{-1}$)* | *Cancer subjects* | *Controls* | *Relative risk*[c] | *Cancer subjects* | *Controls* | *Relative risk*[d] | *Cancer subjects* | *Controls* | *Relative risk*[d] |
| 1st | 2.7–5.8 | 5.28 | 34 | 49 | 1.37 | 26 | 70 | 0.75 | 60 | 119 | 0.99 |
| 2nd | 5.8–6.4 | 6.17 | 21 | 32 | 1.19 | 31 | 55 | 1.08 | 52 | 87 | 1.15 |
| 3rd | 6.4–6.9 | 6.70 | 26 | 42 | 1.14 | 34 | 58 | 1.18 | 60 | 100 | 1.18 |
| 4th | 7.0–7.5 | 7.27 | 19 | 52 | 0.69 | 30 | 57 | 1.04 | 49 | 109 | 0.88 |
| 5th | 7.6–11.0 | 8.25 | 16 | 49 | 0.57 | 30 | 61 | 0.97 | 46 | 110 | 0.81 |
| All | 2.7–11.0 | 6.70 | 116 | 224 | 1.00 | 151 | 301 | 1.00 | 267 | 525 | 1.00 |

[a]Relative risks take into account the matched design of the study and are expressed relative to the risk in the 'all' category; [b]Because the original cholesterol levels were taken to the nearest 0.1 mmol l$^{-1}$, the'quintiles' do not contain equal numbers; [c]Test for trend, $P=0.01$; [d]Test for trend, $P>0.2$.

cancer (<2 years and ≥2 years). For those diagnosed earlier there was a statistically significant declining trend in relative risk from the lowest to the highest cholesterol quintile, but this was not the case for those diagnosed later.

In these data, there was no evidence of an association between serum cholesterol and the age of the subjects and controls. The mean cholesterol was, on average, 0.11 mmol l$^{-1}$ (s.e. = 0.08) higher in current smokers than in non-smokers, but in current smokers of cigarettes alone, there was no evidence of an association between cholesterol and cigarette consumption. Thus the matching for age and smoking habit were less important in this study on serum cholesterol than in our previous studies of serum beta-carotene and vitamin E based on the same series of cancer subjects and controls (Wald *et al.*, 1987, 1988).

### Discussion

In our study the inverse association between serum cholesterol and cancer that others have found was restricted to cancers diagnosed less than 2 years after the date of blood collection. Our data therefore suggest that the low serum cholesterol was a metabolic consequence, rather than a precursor, of the cancer. It follows that a lowered serum cholesterol should not be regarded as a cause of cancer. We have not been able to consider many sites of cancer separately since our study was not large enough to do so.

Most studies, as well as our own, show that the low cholesterol–cancer association is either entirely confined to the short-term (a few years) (Hiatt & Fireman, 1986; Wingard *et al.*, 1984; Salonen, 1982; Wallace *et al.*, 1982; Thomas *et al.*, 1982; Kromhout *et al.*, 1988) or is substantially so; any longer-term association being less marked than the short-term association and no longer statistically significant (Sherwin *et al.*, 1987; International Collaborative Group, 1982; Keys *et al.*, 1985; Gerhardsson *et al.*, 1986; Sorlie & Feinleib, 1982). Some studies, however, show a persistent longer-term association that is of comparable magnitude to the short-term association which is most apparent with lung cancer and, in general, is statistically significant (Kagan *et al.*, 1981; Garcia-Palmieri *et al.*, 1981; Kark *et al.*, 1980; Schatzkin *et al.*, 1987; Morris *et al.*, 1983). The reason for the discrepancy is uncertain, but it is unlikely to be due to chance. The fact that a number of large and well conducted studies show no long-term association between low serum cholesterol and cancer at all sites or cancer at individual sites (including lung) makes it unlikely that the association found in some studies is causal and suggests that it is due to the effect of unidentified confounding factors or other sources of bias present in the studies concerned or the populations examined.

We would like to thank Dr Jillian Boreham for her assistance in data processing.

### References

ALLAIN, C.C., POON, L.S., CHAN, C.S.G., RICHMOND, W. & FU, P.C. (1974). Enzymatic determination of total serum cholesterol. *Clin. Chem.*, **20**, 470.

BRESLOW, N.E. & DAY, N.E. (1980). *Statistical Methods in Cancer Research, Vol. 1, The Analysis of Case–Control Studies*. IARC: Lyon.

GARCIA-PALMIERI, M.R., SORLIE, P.D., COSTAS, R. & HAVLIK, R.J. (1981). An apparent inverse relationship between serum cholesterol and cancer mortality in Puerto Rico. *Am. J. Epidemiol.*, **114**, 29.

GERHARDSSON, M., ROSENQVIST, U., AHLBOM, A. & CARLSON, L.A. (1986). Serum cholesterol and cancer – a retrospective case-control study. *Int. J. Epidemiol.*, **15**, 155.

HIATT, R.A. & FIREMAN, B.H. (1986). Serum cholesterol and the incidence of cancer in a large cohort. *J. Chronic Dis.*, **39**, 861.

HÜNTELER, J.L.A. & VAN DER SLIK, W. (1972). A modification of serum cholesterol determination by continuous flow analysis. *Clin. Chim. Acta*, **42**, 449.

INTERNATIONAL COLLABORATIVE GROUP (1982). Circulating cholesterol level and risk of death from cancer in men aged 40 to 69 years: experience of an international collaborative group. *J. Am. Med. Assoc.*, **248**, 2853.

KAGAN, A., MCGEE, D.L., YANO, K., RHOADS, G.G. & NOMURA, A. (1981). Serum cholesterol and mortality in a Japanese–American population: the Honolulu Heart Program. *Amer. J. Epidemiol.*, **114**, 11.

KARK, J.D., SMITH, A.H. & HAMES, C.G. (1980). The relationship of serum cholesterol to the incidence of cancer in Evans county, Georgia. *J. Chronic Dis.*, **33**, 311.

KEYS, A., ARAVANIS, C., BLACKBURN, H. and 8 others (1985). Serum cholesterol and cancer mortality in the Seven Countries Study. *Am. J. Epidemiol.*, **121**, 870.

KROMHOUT, D., BOSSCHIETER, E.B., DRIJVER, M. & COULANDER, C. DE L. (1988). Serum cholesterol and 25-year incidence of and mortality from myocardial infarction and cancer. *Arch. Intern. Med.*, **148**, 1051.

MORRIS, D.L., BORHANI, N.O., FITZSIMONS, E. and 6 others (1983). Serum cholesterol and cancer in the hypertension detection and follow-up program. *Cancer*, **52**, 1754.

SALONEN, J.T. (1982). Risk of cancer and death in relation to serum cholesterol: a longitudinal study in an Eastern Finnish population with high overall cholesterol level. *Am. J. Epidemiol.*, **116**, 622.

SCHATZKIN, A., HOOVER, R.N., TAYLOR, P.R. and 4 others (1987). Serum cholesterol and cancer in the NHANES 1 epidemiologic follow-up study. *Lancet*, **ii**, 298.

SHERWIN, R.W., WENTWORTH, D.N., CUTLER, J.A., HULLEY, S.B., KULLER, L.H. & STAMLER, J. (1987). Serum cholesterol levels and cancer mortality in 361,662 men screened for the Multiple Risk Factor Intervention Trial. *J. Am. Med. Assoc.*, **257**, 943.

SORLIE, P.D. & FEINLEIB, M. (1982). The serum cholesterol-cancer relationship: an analysis of time trends in the Framingham Study. *J. Natl Cancer Inst.*, **69**, 989.

THOMAS, C.B., DUSZYNSKI, K.R. & SCHAFFER, J.W. (1982). Cholesterol levels in young adulthood and subsequent cancer: a preliminary note. *Johns Hopkins Med. J.*, **150**, 89.

WALD, N., BOREHAM, J. & BAILEY, A. (1986). Serum retinol and subsequent risk of cancer. *Br. J. Cancer*, **54**, 957.

WALD, N., IDLE, M., BOREHAM, J. & BAILEY, A. (1980). Low serum vitamin A and subsequent risk of cancer: preliminary results of a prospective study. *Lancet*, **ii**, 813.

WALD, N.J., THOMPSON, S.G., DENSEM, J.W., BOREHAM, J. & BAILEY, A. (1987). Serum vitamin E and subsequent risk of cancer. *Br. J. Cancer*, **56**, 69.

WALD, N.J., THOMPSON, S.G., DENSEM, J.W., BOREHAM, J. & BAILEY, A. (1988). Serum beta-carotene and subsequent risk of cancer: results from the BUPA Study. *Br. J. Cancer*, **57**, 428.

WALLACE, R.B., ROST, C., BURMEISTER, L.F. & POMREHN, P.R. (1982). Cancer incidence in humans: relationship to plasma lipids and relative weight. *J. Natl Cancer Inst.*, **68**, 915.

WINGARD, D.L., CRIQUI, M.H., HOLDBROOK, M.J. & BARRETT-CONNOR, E. (1984). Plasma cholesterol and cancer morbidity and mortality in an adult community. *J. Chronic Dis.*, **37**, 401.